# Quantifying the population burden of musculoskeletal disorders, including impact on sickness absence: analysis of national Scottish data

**DOI:** 10.1093/rap/rkac030

**Published:** 2022-05-06

**Authors:** Karen Walker-Bone, Helen Storkey, Julie Peacock, Benjamin Ellis, Michael Ly, Jonathan Hill, James O’Malley

**Affiliations:** 1 MRC Versus Arthritis Centre for Musculoskeletal Health and Work; 2 MRC Lifecourse Epidemiology Centre, University of Southampton, Southampton; 3 Public Health Scotland, Edinburgh; 4 Versus Arthritis; 5 Imperial College Healthcare NHS Trust, London; 6 Primary Care Centre Versus Arthritis, School of Medicine, Keele University, Keele, UK

**Keywords:** musculoskeletal disorders, burden, systematic data, prevalence, sickness absence, deprivation

## Abstract

**Objectives:**

Musculoskeletal disorders (MSDs) account for the greatest burden of years lived with disability globally. To prevent disability, good-quality services need to be commissioned, appropriate for local need. We analysed data collected systematically from a new musculoskeletal service serving 70% of the population of Scotland to evaluate: age- and sex-specific occurrence; anatomical distribution; and impact and effect on work ability.

**Methods:**

A new centralized telephone-based triage for people with musculoskeletal disorders was set up in Scotland in 2015. Available to most of the population aged >16 years (>3 million people), data were collected systematically into a database detailing: anatomical site, nature of onset, duration, impact/risk (modified STarT score), deprivation level and, for those in employment, sickness absence.

**Results:**

Data were available from 219 314 new callers, 2015–18. Calls were more frequently from women (60%), increased with age until the eighth decade, and 66% reported symptoms that had been present for >6 weeks. Callers were more likely to be living in more deprived areas in each age band between 20 and 64 years and tended to have higher-impact symptoms. The majority (53%) of callers were in employment, and 19% of these were off sick because of their symptoms. Sickness absence was more common among those with highest impact/risk scores from deprived areas with more acute symptoms.

**Discussion:**

Large-scale systematic data collection for MSDs emphasizes the size and impact of the burden among adults aged >16 years. A socio-economic gradient is evident in terms of prevalence and impact of MSDs, particularly for sickness absence.

Key messages

- Systematic data collection about musculoskeletal disorders facilitates targeted local prevention strategies and care pathways.
- Deprivation is associated with a greater prevalence of, and impact/risk from, musculoskeletal disorders.
- Musculoskeletal disorders cause substantial sickness absence, and there is a socio-economic gradient.

## Introduction

Musculoskeletal disorders (MSDs) are the most significant contributors to disability worldwide [1], causing ≥17% of years lived with disability [2]. Health-care costs for MSDs are massive (among the top five costliest of all conditions classified by the International Classification of Diseases) [3]. Moreover, it is widely predicted that the prevalence and impact of MSDs will increase [4], as a result of population ageing, increasing prevalence of other non-communicable diseases and their modifiable risk factors (e.g. obesity), and increasing rates of fractures associated with bone fragility, falls and road traffic accidents. Consequently, finding ways to prevent disability from MSDs is a major and important challenge [5].

In the UK, pathways of care for MSDs have not always been clear or effective, leading to avoidable costs and poor patient and work outcomes [6, 7], despite an annual spend exceeding £5 billion in England (population 56 million) [5] and £353 million in Scotland (population 6 million) [8]. Even so, Scottish data from 2016 showed that low back and neck pain were the second largest cause of years lived with disability (totalling 67 900 years) [9]. There is considerable evidence about what works to improve musculoskeletal health [10]. However, to provide comprehensive, effective services for the prevention and treatment of MSDs, health-care providers and policy-makers need local data about the prevalence of these conditions and their impact and risk factors. Data collection in routine musculoskeletal services has been found to be incomplete, unstandardized and non-systematic [5]. Although useful data are available from primary care databases, such as the Clinical Practice Research datalink (CPRD), they provide no information about impact/risk, and there are a number of methodological and coding issues, which particularly hamper interpretation of data about some of the most common conditions (e.g. regional pain disorders and OA) [11, 12].

UK health-care services were devolved in 2015–16, giving individual countries opportunities to control their budgets and prioritize service provision. In Scotland, a new triage service for musculoskeletal symptoms was incepted, serving the majority of the adult population, which created an opportunity to analyse large-scale systematically collected data from new calls over 3 years to gain a better understanding of the age- and sex-specific occurrence, anatomical distribution, impact/risk status and effect on work, taking account of levels of population deprivation.

## Methods

A new centralized telephone triage service (the MSK helpline) was introduced in 2015 for people aged >16 years with MSD symptoms in Scotland. It was advertised as the first point of contact for people experiencing symptoms of MSDs (e.g. back pain and sports injuries) through general practitioner (GP) surgeries, health boards and online. In some areas, people with MSDs could obtain musculoskeletal health care only if they contacted the helpline, but more latitude was seen in other areas. Operated by the Musculoskeletal Advice and Triage Service, calls were answered by trained operators, supported by nurses and physiotherapists. Information was collected systematically using a pre-defined script, which initially screened for signs of abdominal aortic aneurysm, deep vein thrombosis and cauda equina syndrome (all referred urgently to a GP if screening questions were positive). Subsequent high-level musculoskeletal screening questions were asked, covering symptoms consistent with the red flags [13], and in the event of positive responses, the call was transferred to a clinically trained member of staff. For everyone else, questions were asked about their current main MSD: its anatomical site; its duration (<1, 1–2, 2–3, 4–6 or 6–12 weeks or >3 months); pattern of onset (gradual onset without specific trigger; accident/injury; a sudden onset without specific trigger; had pain off and on for a long time) and whether or not it was recurrent. They were also asked if they were currently working, and if yes, whether they were off sick because of their symptoms. In Scotland, health services are delivered through 14 geographical health boards. At the time of data collection, this service was available to people living in areas covered by 9 of the 14 Scottish Health Boards, serving a population of 3.17 million people aged >16 years out of the total 4.52 million >16 years (70% of the total) resident in Scotland.

Every caller was asked nine questions. Seven of these were very close to the questions in the validated STarT Back tool [14, 15] but were modified to be asked by the telephone operator instead of self-reported and to be relevant to musculoskeletal pain at any site, rather than only the back (wording ‘back pain’ altered to ‘pain’). Two questions were additionally modified so that ‘pain has spread down my leg’ was altered to ‘pain in more than one part of the body’ and ‘pain in the shoulder or neck’ was modified to ‘has the most painful area been in your hand, wrist or elbow?’. Therefore, the questions explored, in relationship to symptoms over the past 2 weeks: functional impact; pain at more than one site; beliefs about pain and activity; worrying thoughts; lack of enjoyment; catastrophization and bothersomeness (options: not at all; slightly; moderately; very much; extremely). Based upon their responses, the caller was triaged as low (total score ≤ 3), medium (total score ≥ 4 and sub-score from questions 5–9 < 3) or high risk (total score > 4 and sub-score ≥ 4). Generally, callers with a low risk score were triaged to information to support self-management, whereas those with medium or high risk scores were offered referral or requested to make an appointment with their GP.

The Scottish index of multiple deprivation (SIMD) 2016 uses updated 2011 Census data to produce an area-based relative measure of deprivation. The index takes into account seven domains: income, employment, education, health, access to services, crime and housing. It has been calculated and ranked for 6796 areas of Scotland (data zones), each of which includes on average 760 people. The SIMD quintiles split the ranked data zones into five groups, each containing 20% of Scotland’s data zones (quintile 1 = most deprived). The SIMD was calculated from the postcode of each new caller.

This research was carried out in accord with the Declaration of Helsinki. Anonymized, routinely collected data were analysed. Approval for the analysis and write-up were attained from NHS 24 (23 September 2020).

### Statistical analysis

Data about all new callers to the MSK helpline between 2015 and 2018 were analysed. Descriptive statistics were used to report the age- and sex-specific rates of new callers per 1000 population, the reported duration of the main symptom and pattern of onset. Rates of calls per 1000 for the anatomical site of the main symptom were also described by age and gender. Rates of new calls to the helpline per 1000 population were summarized graphically by quintile of deprivation. A modified STarT score was assigned to the main musculoskeletal symptom of each new caller, and these scores were then summarized graphically for men and women by quintile of deprivation. The STarT Back scoring system has been validated in various settings for people with back pain [14, 16–18], and therefore, the range of STarT scores by deprivation quintile for people whose main problem was back pain (with/without leg pain) was also explored. Among those currently working, the proportion off sick because of their MSD was calculated, and these data were presented in relationship to the duration of symptoms and stratified by the modified STarT score. Finally, the proportion of employed callers off sick was summarized by symptom duration and quintile of deprivation.

The de-identified analyses were carried out within NHS 24, NHS Scotland, and permission was granted for us to publish the data by the owners, NHS 24 Service delivery team, NHS Scotland, 28 September 2020.

## Results

**Fig. 1:**
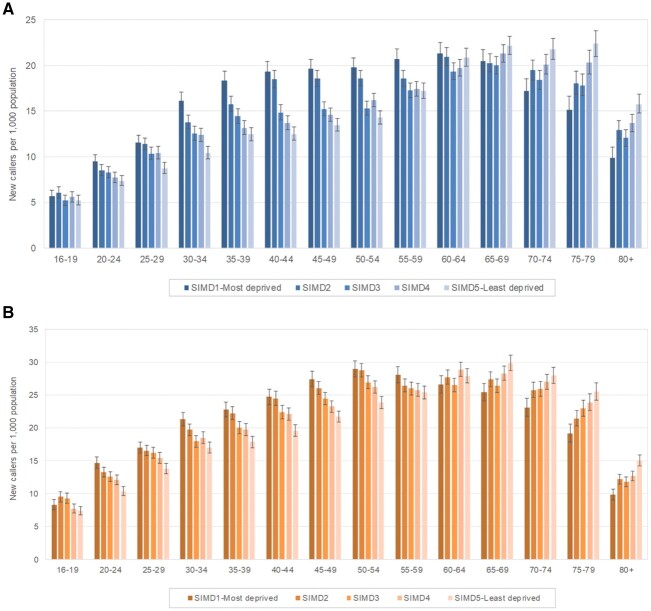
Rates of new calls to the MSK helpline during 2015–18 per 1000 population by quintile of deprivation for men (**A**) and women (**B**) SIMD: Scottish index of multiple deprivation.

A total of 302 045 calls were made to the MSK helpline in 2015–18. After exclusion of invalid calls, repeat callers, duplicate records or calls for whom essential data fields were missing, data were available for analysis from 219 314 new calls (73%). Around 50 000 calls were received annually (range: 50 481–63 213) from ∼1.7% of the eligible population. More calls were made by women (60%) than men (40%) at all ages. Fig. 1 summarizes the rates of new calls by age and quintile of deprivation. The numbers of calls increased by age band until a peak at age 50–54 years in women and 55–59 years in men. With the exception of the youngest age group (16–19 years), there was a consistent trend for more calls from people living in more deprived areas until age 60–64 years, after which the opposite was observed, and calls were more common from those living in less deprived areas. Among men, for example, 12% of the callers aged >60 years were from the most deprived quintile compared with 23% from the least deprived quintile.

Table 1 summarizes the rates of new calls by anatomical site of the main musculoskeletal symptom and age. Back pain (with/without leg pain) was the most common [*n* = 62 956 (29%) calls] and shoulder pain the next most common [37 644 (17%) calls], followed by knee pain [33 683 (15%) calls]. Elbow, ankle and foot made up >50% of the calls labelled as ‘other joint’ (∼17 000 calls). Three thousand and fifty-one (1.4%) callers wanted to access a walking aid or splint. Hip and shoulder symptoms were more common with increasing age. Most common in the youngest age group were symptoms in the back (39%) and knee (18%). Supplementary Table S1, available at *Rheumatology Advances in Practice* online, shows the rates of new calls by gender and anatomical site: women reported MSDs at all sites more commonly than men, but the anatomical sites affected were proportionately similar.

**Table 1:** Anatomical distribution of current musculoskeletal problem reported to MSK helpline by age group

Body site	16–29 years		30–39 years		40–49 years		50–59 years		60–69 years		≥70 years		All ages (≥16 years)	
	*n*	Percentage of total	*n*	Percentage of total	*n*	Percentage of total	*n*	Percentage of total	*n*	Percentage of total	*n*	Percentage of total	*n*	Percentage of total
Shoulder(s)	2678	10	3742	12	7006	18	9486	21	8095	19	6637	19	37 644	17
Back only	6966	26	6881	22	5901	15	5687	12	4648	11	4091	12	34 174	16
Back + leg(s)	3376	13	4861	16	5275	14	5587	12	5222	13	4461	13	28 782	13
Neck only	666	2	875	3	1161	3	1417	3	1530	4	1693	5	7342	3
Neck + arm(s)	804	3	1538	5	2395	6	2887	6	2402	6	1774	5	11 800	5
Knee(s)	4908	18	4024	13	4876	13	6897	15	7243	17	5735	16	33 683	15
Other limb/joint(s)	4185	16	4737	15	6763	18	7667	17	5833	14	4359	12	33 544	15
Hip(s)	1441	5	1620	5	2023	5	2966	6	3688	9	3727	10	15 465	7
Other	1675	6	2073	7	2271	6	2824	6	2492	6	2175	6	13 510	6
Walking aid	198	1	259	1	465	1	663	1	616	1	850	2	3051	1
All body sites	26 897		30 610		38 136		46 081		41 769		35 502		218 995	

The majority (66%) of new callers had experienced symptoms for >6 weeks and 50% for >3 months before calling. Symptom duration did not vary by age band, gender, anatomical site or calendar year (data not shown). When asked about the pattern of symptom onset, the commonest response was gradual onset without specific trigger (30%); 24% ascribed their symptoms to accident/injury; 25% reported sudden onset without specific trigger; and the remainder (20%) reported pain off and on for a long time. The age group in which accidents and injuries were most commonly reported as the cause were those aged <40 years, but there was another smaller increase in accidents/injuries among those aged >70 years compared with those aged 60–69 years.

Using the modified STarT scoring system, >52 000 callers were identified as low risk (24%), almost 77 000 (35%) as medium risk and the remaining 90 000 (41%) as high risk. As mandated, most of those in the low-risk group received advice to self-manage (73%) or advice to self-manage with a referral (14%), 7% received ‘other’, and 6% were provided with a walking aid or splint. In contrast, 98% of those in the medium- and high-risk groups received onward referral, with only 2% advised to self-manage or being provided with a walking aid/splint.

**Fig. 2:**
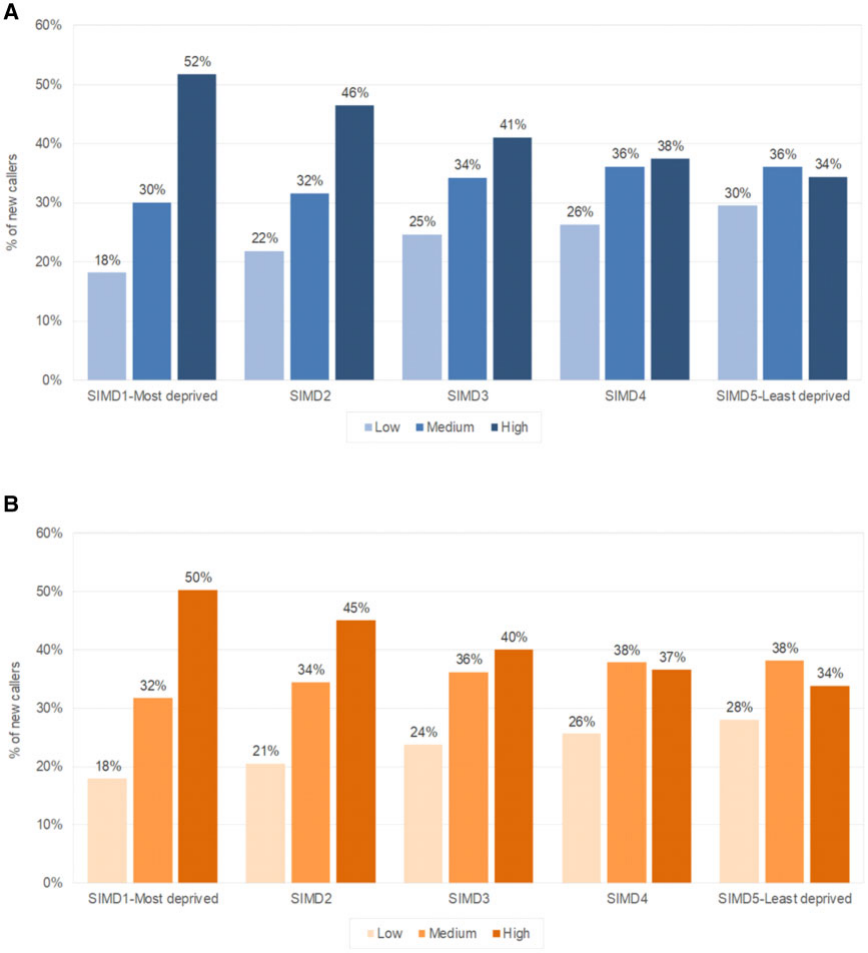
Summary of modified STarT scores by Scottish index of multiple deprivation quintiles of deprivation among men (**A**) and women (**b**) SIMD: Scottish index of multiple deprivation.

**Fig. 3:**
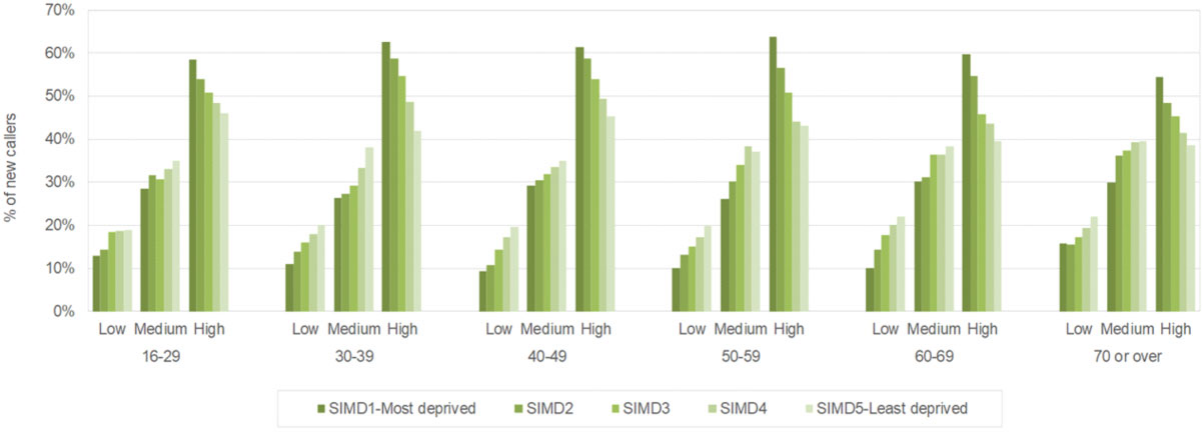
STarT Back scores for new callers to the MSK helpline with back pain (with or without leg pain) by age band and quintiles of deprivation (men and women combined) SIMD: Scottish index of multiple deprivation.

Fig. 2 summarizes the modified STarT scores by quintiles of SIMD. A clear gradation was seen, such that those in the three most deprived quintiles among men and women were considerably more likely to have the highest risk scores. In contrast, approximately one-third of those in the least deprived quintile had high, medium and low risk scores. These relationships are also shown in Fig. 3, in which the STarT scores among callers reporting back pain (with or without leg pain) are summarized by age and quintiles of deprivation (men and women combined). No matter the age or gender of the caller, more calls about back pain were made from people living in the most deprived areas.

In total, 116 116 (57%) new callers reported current employment (55% of women and 59% of men). Confining to those of traditional working age, 75.5% of women aged 25–59 years and 76.9% of men aged 25–64 years were working. Among these, 22 191 (19%) of callers were off sick because of their MSD. Table 2 summarizes the proportions of people off sick (for those in employment), stratified by modified STarT score and duration of MSK symptoms. Rates of sickness absence increased with modified STarT score, such that one in four workers with high risk scores were currently off sick because of their MSD. Rates of sickness absence were generally higher among those with more recent-onset symptoms (46% of those in employment with MSK problem <1 week). However, 12% of employed callers reported sickness absence with symptoms that had been present for >3 months.

**Table 2:** Rates of sickness absence by modified STarT score and by duration of musculoskeletal problem among employed callers to the MSK helpline

	Men			Women			All		
Modified STarT score	Number in work	Number off sick	Percentage off sick	Number in work	Number off sick	Percentage off sick	Number in work	Number off sick	Percentage off sick
Low risk	10 633	1039	9.8	14 849	1037	7.0	25 482	2076	8.1
Medium risk	16 954	3403	20.0	25 106	4162	16.6	42 060	7565	18.0
High risk	20 227	5825	28.8	26 407	6557	24.8	46 634	12 382	26.6
Duration of musculoskeletal problem									
<1 week	3319	1643	49.5	3941	1670	42.4	7260	3313	45.6
1–2 weeks	3485	1397	40.0	4641	1575	33.9	8126	2972	36.6
2–3 weeks	6320	1876	29.7	8444	2193	26.0	14764	4069	27.6
4–6 weeks	4563	1048	23.0	6253	1237	19.8	10816	2285	21.1
6–12 weeks	8217	1326	16.1	10962	1588	14.5	19179	2914	15.2
>3 months	21910	2977	13.6	32121	3493	10.9	54031	6470	12.0

**Fig. 4:**
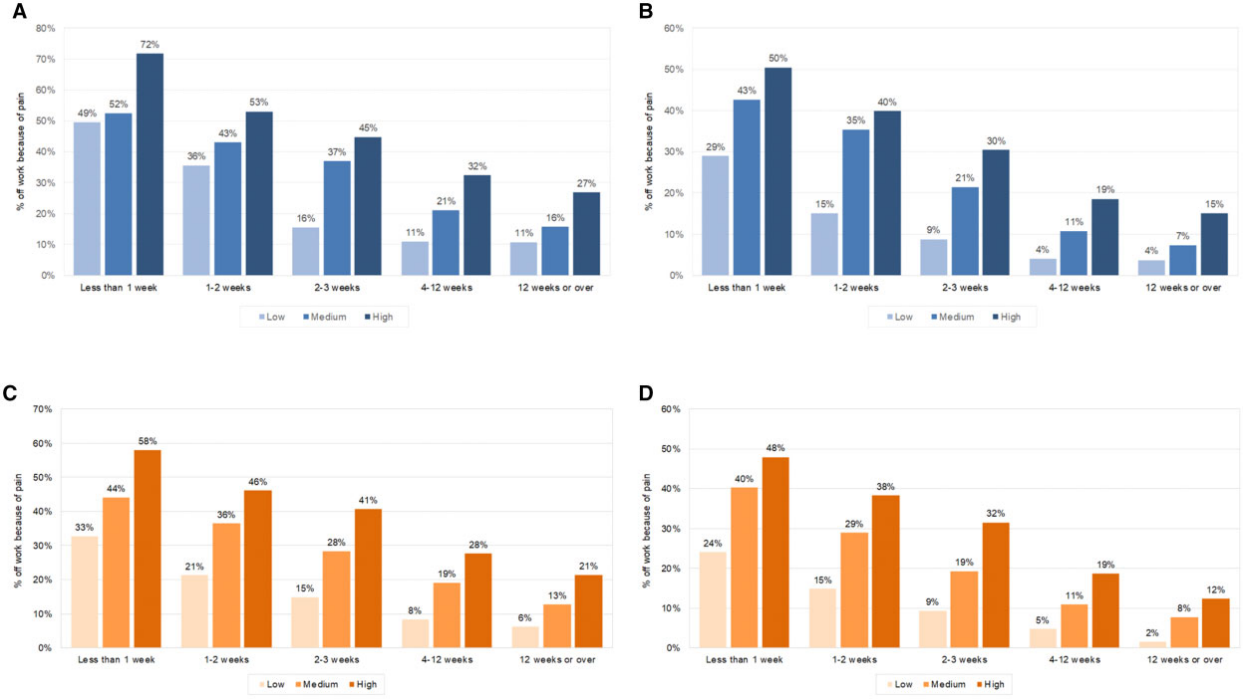
Comparison of the proportion of men and women reporting pain-induced sick leave in relationship to duration of symptoms and modified STarT score among those in the SIMD quintiles 1 and 5 (**A**) Men, SIMD1 (most deprived). (**B**) Men, SIMD5 (least deprived). (**C**) Women, SIMD1 (most deprived). (**D**) Women, SIMD5 (least deprived). SIMD: Scottish index of multiple deprivation.

Fig. 4 shows, by gender, the rates of sickness absence associated with high- medium- and low-risk modified STarT scores, comparing those in SIMD1 (most deprived) with those in SIMD5 (least deprived). Although employment rates were lower among people living in more deprived areas, higher rates of sickness absence were reported by workers in SIMD1, with effects apparently greater among men (72 *vs* 50% off sick with highest risk scores and duration of symptoms < 1 week) than women (58 *vs* 48% off sick with highest risk scores and duration of symptoms < 1 week).

## Discussion

This analysis of systematically collected data from new callers to the MSK helpline provides insight about the size of the burden of MSDs in a defined adult population of 3.2 million people (>70% of the total) in Scotland. In total, over 3 years, 1.7% of the eligible population made a new call. More calls were made by women than men (60 *vs* 40%), and the commonest symptom was back pain (with/without leg pain). Most callers reported long-term symptoms (66% >6 weeks and 50% >3 months). Grading impact/risk using a modified STarT score [14, 15], only a minority of callers (24%) were defined as low risk, and the largest group (41%) was high risk. Considering the STarT scores alongside SIMD showed a consistent relationship between higher risk scores and living in a more deprived area among male and female callers up to age 65 years, but in older callers, the opposite relationship was seen. Confining the analysis only to callers with back pain and using the STarT Back scoring system as validated [14–19], similar relationships were seen between highest risk scores and deprivation throughout the age range. Slightly more than half of callers were in employment, and of these, almost one in five (19%) was currently off sick because of their MSD. Sickness absence was more common among those with a shorter duration of symptoms (particularly <1 week), but regardless of the duration of symptoms, it was consistently more common among those with high-risk modified STarT scores and among people living in more deprived areas.

These data must be considered alongside some limitations. Musculoskeletal pain is known to be highly prevalent in the general population, and there are multiple ways in which people can access primary care for MSDs. For example, some patients might have chosen to see their GP or private provider or to attend accident and emergency services, rather than use the telephone helpline. It is clear, therefore, that 220 000 calls from new callers over 3 years from 3 million people will not be capturing all people with MSK symptoms who were seeking care. In addition, the helpline was not adopted simultaneously across the whole of Scotland in 2015, and some of the health boards incepted the service during the period of data collection. Therefore, the data are presented per 1000 population who had access to the service at each point in time. However, not only was the commissioning of the service variable by health board, but so was the method of dissemination or publicizing of the helpline. In some health boards, the service was implemented such that people with MSDs could obtain musculoskeletal health care only if they contacted the helpline, but this was not the case everywhere. Therefore, although the denominator is accurate in terms of exactly which population groups were able to access the service, these will be relative under-estimates of the real demand. Notably, because Greater Glasgow and Clyde was one of the five health boards that did not commission this service, the total adult population living in SIMD1 (most deprived) was slightly under-represented (15% of the population were in SIMD1 in these analyses *vs* 19% for the entire Scottish population). Importantly, this analysis focused only on new callers (73% of total calls). Repeat callers might be more likely to have long-term conditions, chronic pain or more troublesome symptoms, and it is important to bear in mind, therefore, that the data presented here represent only a tip of the iceberg. The Commissioners of the Scotland MSK helpline chose to adapt the STarT Back tool to make it suitable for callers with any type of musculoskeletal pain condition. Although STarT Back has been well validated and widely used, this modified tool has not been validated. However, colleagues at the University of Keele have recently developed and validated the Keele STarT MSK tool, with 10 questions aiming to rate risk of poor outcomes in three categories (low, medium and high), creating a valid tool similar to that used here [20]. There were some missing data from the helpline. For these analyses, calls missing a new caller status were excluded, but we included all other calls. For most variables, few data were missing (<5%), but in 2018, one health board elected to stop asking about employment, and this resulted in 13% of all callers that year having missing data about employment status and sick leave. In consequence, the rates of sickness absence attributable to MSDs presented here are likely to be an under-estimate, although we do not believe that this will have had a selective effect on the rates of sickness absence by SIMD. Finally, area-level deprivation scores, such as SIMD, can be criticized because not every person living in any one area will be the same. Socio-economic position varies widely depending upon pre- and post-natal environment and parental circumstances, in addition to the domains captured and summarized in SIMD. Reassuringly, one US study of relocations found that 78% of people moved to a neighbourhood in a similar deprivation quintile, with only a 2–13% chance that an individual moved outside their quintile annually [21]. However, clearly the 760 people living in one area cannot all be the same. Of course, this limitation would tend to push our findings towards the null hypothesis (that deprivation was not important); therefore, it is striking that we have found the trends summarized here with quintiles of deprivation.

The finding that such a high proportion of callers were graded as high risk according to the modified STarT tool was interesting and unexpected when compared with findings from other studies, in which the largest group are usually low risk [14–19]. Of course, the tool was modified in its administration/questions, and this might have impacted our findings. Certainly, for this population-based screening tool, the developers were aiming not to reassure too many callers inappropriately. However, another possibility is that people with more trivial symptoms trying to access care do not choose to telephone the helpline and opt instead to self-manage their symptoms or access care privately or choose complementary or alternative health care.

Although a social gradient was not unexpected, it is interesting that the social gradient of calls appeared to switch at around age 65 years (more calls from least deprived quintiles >65 years). It could be that this is explained by higher rates of mortality among those from deprived backgrounds, or that older people from deprived areas are less aware of, or less able to access, this service. An alternative explanation might be that individuals with higher levels of deprivation have already been identified elsewhere in the health-care system as high risk and been referred through other channels for care (e.g. pain clinics, elderly medicine, orthopaedics or rheumatology). Another hypothesis is that after retirement, social factors become less important and biological factors more important, or that inequalities at older ages are more effectively narrowed by welfare programmes and/or social policies [22]. However, the cumulative inequality theory would suggest that rates of inequality increase throughout the life course as risk factors accumulate [23, 24]. Interestingly, Swedish researchers who explored the effects of age, socio-economic factors and birth cohort on pain, distress and dental health found similar results for pain [25]. Their analysis showed that, although relative inequalities declined in later life (>75 years), absolute inequalities remained substantial, and that cumulative disadvantage continued to drive differences up to 45–64 years, but beyond this, factors related to ageing started to impact in the opposite direction, thereby somewhat reducing the socio-economic gap [25].

That there is a socio-economic gradient in MSDs is not a new finding. Chronic pain, for example, is more prevalent and burdensome among people with poorer socio-economic circumstances [26]. Back pain has been found to be more disabling among less well-educated people [27] and more intense with less advantaged job position [28]. Moreover, people with RA and other chronic musculoskeletal conditions having poorer educational attainment were found to have two to three times higher mortality rates [29, 30]. Likewise, higher rates of mortality were found among white people aged 25–64 years with SLE with poorer educational attainment [31]. However, rarely are data available for the whole breadth of MSDs for a population >3 million people. The socio-economic gradient shown here both for rates of new calls to the helpline and for impact according to the modified STarT score is striking. Moreover, although rates of employment were lower among those from more deprived areas, rates of sickness absence caused by MSDs were higher. This finding is important because, at least among people off sick with low back pain, there was an important association between the duration of absence and the chances of ever working again: people off sick for <4 weeks had a 93% chance of returning, whereas people absent for >6 months had a 68% chance of ever returning to work [32]. Employment has a pivotal role in reducing health inequalities [33], and unemployment is associated with poorer health, increased risk of self-harm and suicide and increased health-care needs [34–38]. For this reason, early intervention among people off sick with MSDs is emphasized [39]. According to our results, >22 000 people were off sick with MSDs in Scotland during 2015–18, and 12% of these reported an MSD >3 months in duration, with a social gradient in sickness absence. There are two possible explanations for this. Firstly, physically demanding jobs have been found to increase the risk of consultation for MSDs [40]. Secondly, people with poorer educational attainment are more likely to be employed in physically demanding jobs (e.g. construction, manufacturing) and could find themselves more work-disabled by a painful MSD than an individual whose job is sedentary and who has some flexibility and/or autonomy at work. Overall, these analyses suggest a substantial need for services to prevent MSDs and, where necessary, deliver tailored, prompt, evidence-based treatment, targeted to the most deprived areas, not only to improve health, but also to enable employment, reduce inequalities and save health and welfare costs.

In summary, by analysis of systematically collected data, we have found effects of age and gender but also a socio-economic gradient, not only for prevalence but also for impact, including sickness absence from work.

*Funding:* This work was supported by Versus Arthritis on behalf of the MRC Versus Arthritis Centre for Musculoskeletal Health and Work (ref.: 22090).

*Disclosure statement:* The authors have declared no conflicts of interest.

## Data availability statement

These data are not currently freely available. Researchers interested in accessing the data would need to apply to NHS 24.

## Supplementary data

Supplementary data are available at *Rheumatology Advances in Practice* online.

## Supplementary Material

rkac030_Supplementary_Data
