# Supplementary material for: Quantifying the population burden of musculoskeletal disorders, including impact on sickness absence: analysis of national Scottish data
Source: Rheumatol Adv Pract. 2022 May 6;6(2):rkac030. doi: 10.1093/rap/rkac030 (PMC9113281; doi:10.1093/rap/rkac030)
Supplement: rkac030_Supplementary_Data [file rkac030_supplementary_data.docx]

**Supplementary Table S1. Rates of new calls to MSK helpline 2015-18 by anatomical site of current musculoskeletal disorder for men and women.**

| **Body Site (Main problem calling about)** | **Men** | | **Women** | |
| --- | --- | --- | --- | --- |
|  | **Number** | **% of  Total** | **Number** | **% of  Total** |
| Back only | 14,064 | 16% | 20,110 | 15% |
| Back and one leg | 7,971 | 9% | 12,801 | 10% |
| Back and both legs | 2,776 | 3% | 5,234 | 4% |
| Neck only | 3,022 | 3% | 4,320 | 3% |
| Neck and one arm | 3,230 | 4% | 5,770 | 4% |
| Neck and both arms | 955 | 1% | 1,845 | 1% |
| Shoulder - Single | 15,026 | 17% | 19,571 | 15% |
| Shoulder - Bilateral | 1,460 | 2% | 1,587 | 1% |
| Knee - Single | 12,029 | 14% | 14,785 | 11% |
| Knee - Bilateral | 2,652 | 3% | 4,217 | 3% |
| Hip - Single | 3,936 | 5% | 8,645 | 7% |
| Hip - Bilateral | 738 | 1% | 2,146 | 2% |
| Other limb/joint – Single (elbow, wrist/hand ankle, foot) | 11,209 | 13% | 16,763 | 13% |
| Other limb/joint – Both (elbow, wrist/hand, ankle, foot) | 1,964 | 2% | 3,608 | 3% |
| Other | 4,826 | 6% | 8,684 | 7% |
| Walking aid | 1,090 | 1% | 1,961 | 1% |
| **All body sites** | **86,948** |  | **132,047** |  |

Footnote: Final number is not 219,314 as this information was missing from some calls
